# Can Gait Signatures Provide Quantitative Measures for Aiding Clinical Decision-Making? A Systematic Meta-Analysis of Gait Variability Behavior in Patients with Parkinson's Disease

**DOI:** 10.3389/fnhum.2016.00319

**Published:** 2016-06-30

**Authors:** Niklas König, Navrag B. Singh, Christian R. Baumann, William R. Taylor

**Affiliations:** ^1^Department of Health Sciences and Technology, Institute for Biomechanics, Swiss Federal Institute of Technology in Zurich (ETHZ)Zürich, Switzerland; ^2^Department of Neurology, University Hospital Zürich, University of ZürichZürich, Switzerland

**Keywords:** gait variability, walking balance, dynamic stability, systematic review, meta-analysis, quality of movement, rhythmicity

## Abstract

A disturbed, inconsistent walking pattern is a common feature of patients with Parkinson's disease (PwPD). Such extreme variability in both temporal and spatial parameters of gait has been associated with unstable walking and an elevated prevalence of falls. However, despite their ability to discretise healthy from pathological function, normative *variability* values for key gait parameters are still missing. Furthermore, an understanding of each parameter's response to pathology, as well as the inter-parameter relationships, has received little attention. The aim of this systematic literature review and meta-analysis was therefore to define threshold levels for pathological gait variability as well as to investigate whether all gait parameters are equally perturbed in PwPD. Based on a broader systematic literature search that included 13′195 titles, 34 studies addressed Parkinson's disease, presenting 800 PwPD and 854 healthy subjects. Eight gait parameters were compared, of which six showed increased levels of variability during walking in PwPD. The most commonly reported parameter, coefficient of variation of stride time, revealed an upper threshold of 2.4% to discriminate the two groups. Variability of step width, however, was consistently lower in PwPD compared to healthy subjects, and therefore suggests an explicit sensory motor system control mechanism to prioritize balance during walking. The results provide a clear functional threshold for monitoring treatment efficacy in patients with Parkinson's disease. More importantly, however, quantification of specific functional deficits could well provide a basis for locating the source and extent of the neurological damage, and therefore aid clinical decision-making for individualizing therapies.

## Introduction

Disturbance of normal walking patterns, caused by symptoms such as akinesia and loss of postural reflexes, is a well-acknowledged problem in patients with Parkinson's disease (PwPD) (Hausdorff, 2009). With the progression of the disease, balance, gait, and mobility are increasingly impaired, causing a loss of independence, and consequently a reduction in the quality of life (Damiano et al., 1999; Ellis et al., 2011). This loss of function, mobility, and independence is associated with further complications such as cognitive impairments, sleep disorders (Imbach et al., 2012), depression, cardiovascular diseases (Ton et al., 2010, 2012), and injuries and fatalities (Balash et al., 2005). Despite established pharmaceutical and surgical therapies for treating motor symptoms in PwPD, the disease poses immense challenges for clinicians to identify the disease onset at an early time point, provide a long-term objective evaluation and monitoring of therapies, but also to quantify differences between therapies.

Although, a number of recognized biomarkers for the clinical identification and evaluation of Parkinson's disease (PD) exist (Andersen et al., 2016; Salat et al., 2016), objective methods to measure human movement have become increasing available, and now provide the potential to complement clinical decision-making (Lord et al., 2011a, 2013). In an attempt to translate parameters derived from kinematics into an understanding of movement quality, several measures of both spatial and temporal gait have been investigated: summary measures based on the statistical mean (e.g., mean stride length; Faist et al., 2001; Ferrarin et al., 2005; Hausdorff, 2009), measures to quantify variability during walking based on the standard deviation (e.g., coefficient of variation of stride length; Blin et al., 1990, 1991; Hausdorff et al., 1998; Baltadjieva et al., 2006; Roemmich et al., 2012), measures to quantify bilateral symmetry of walking (e.g., phase coordination index; Plotnik et al., 2007; Plotnik and Hausdorff, 2008; Johnsen et al., 2009; Fasano et al., 2011), and non-linear algorithms that evaluate the structure of gait signals in relation to their temporal evolution (e.g., Lyapunov exponent; Dingwell and Cusumano, 2000; Bruijn et al., 2013; Roemmich et al., 2013). However, since PD is known to disturb rhythmicity of walking (constancy of step repetitions), mean measures for assessing degeneration of this temporal parameter simply result in averaging out any modifications and are therefore entirely insensitive. As a result, only parameters that highlight non-constancy over multiple repetitions can provide objective interpretation of modifications that occur with degeneration due to PD. While non-linear and bilateral symmetry measures of walking provide promising candidates for evaluating such degeneration, due to their simple implementation, parameters of gait variability have so far received the most attention (Hamacher et al., 2011; Lord et al., 2011a; Konig et al., 2014a). However, until now, thorough evaluation of measures of variability for understanding the quality of movement remains missing, and in particular, which threshold levels of gait variability can be considered physiological vs. pathological.

Generally, motor variability increases with aging and pathology while functional performance decreases. As a consequence, variability has been assumed to be detrimental for task performance (Hamacher et al., 2011; König et al., 2016). Traditionally, motor variability was thought to be the result of noisy signaling processes within the human sensory motor system (HSMS). However, recently it has been shown that motor variability can be adapted depending on the motor task requirements, and can also play an important role for successful motor learning (Roerdink et al., 2006; Dingwell and Kang, 2007; Wilson et al., 2008; Russell and Haworth, 2014; Wu et al., 2014; Pekny et al., 2015). As a result, it appears that motor variability is an integral aspect of human movement as well as a prerequisite for effective task performance. It has been argued that excessively high levels of variability render task performance unstable, whereas extremely low levels result in rigid motor performance, hampering the subject's ability to respond to changing environmental conditions. As a result, it seems plausible that an optimal level of variability for successful task performance exists (Todorov and Jordan, 2002; Stergiou et al., 2006). Indeed, in a large systematic review of the literature, it has recently been revealed that an optimal *window* of variability during walking and balancing exists, and which was somewhat consistent across a variety of neuromotor pathologies including stroke, brain injury, and disorders of the basal ganglia etc (König et al., 2016). However, while a consistent window of optimal levels of variability of stride time was presented, it remains unknown whether all parameters of variability respond in a similar manner to degenerated motor control in extrapyramidal diseases.

An efficient walking pattern is characterized by a plethora of concepts such as rhythmicity, regularity, bilateral, and inter-segment coordination, balance control during one-legged and double limb support phases, and controlled forward progression etc., as well as the maintenance of boundary constraints such as sufficient toe clearance to avoid obstacles (Lord et al., 2013). However, such complex concepts to represent gait quality cannot be readily captured in any single parameter, and also not described in terms of quantity (less may not necessarily be better). An additional difficulty in quantifying the degeneration of gait quality due to pathology is that gait is controlled by the coordinated action of various central and peripheral neural circuits (Dietz, 1992, 2002, 2003; Arshavsky et al., 1997; Rosano et al., 2008). As a result, the primary understanding of many neural control mechanisms until now has come from clinical observations of various gait abnormalities (e.g., gait in hypokinetic vs. hyperkinetic disorders) in patients with known neuro-motor diseases, rather than an objective quantification of parameter deviations. For example, an observation of reduced step length in PwPD does not permit conclusions regarding the quality of walking, nor is it indicative of the underlying neural deficit. Importantly, these subjective observations generally lack sensitivity for the early identification of subtle and/or emerging pathologies.

In order to enable improved interpretation of gait metrics in clinical settings for diagnosis and assessment of gait quality and thus treatment effects in PwPD, the aim of this systematic literature review and meta-analysis was therefore to (1) establish whether different gait parameters are indicative of pathological disturbances of the HSMS in PwPD and (2) to define clear threshold values for healthy and pathological gait variability.

## Methods

### Literature search and selection

The data presented here comprises a follow-up analysis of a wider systematic review that compared motor variability in patients with various neurological diseases against the levels observed in asymptomatic subjects (König et al., 2016). While it achieved a new perspective on the effect of pathology on stride time variability, providing for the first time a window of optimal variability, it did not address the distinct effects of pathology on different measures of gait. In this current study, we therefore focus specifically on literature assessing measures to quantify variability during walking based on the standard deviation in PwPD.

The original systematic literature search was conducted with the aim to comprehensively identify studies in which measures of motor variability during walking and standing were collected in both a cohort of healthy elderly and a cohort of patients with a neurological pathology (König et al., 2016). There, a common search string was entered into four different databases (Pubmed, ISI Web of Knowledge, Embase and Ebsco). The search string contained Boolean operators such that an AND-combination of terms specified the *task (*e.g., walk^*^), measure (e.g., *variability)*, and *cohort (*e.g., Parkin^*^). Within these categories synonyms as well as specifications of additional pathological cohorts were combined using the OR operator. The search was limited to original research articles published after the year 1980. Initially, the search revealed 13′195 publications potentially relevant for addressing the question of motor variability across neural pathologies. In two steps, eligibility of studies was assessed using the double-screening method (NK & NS) firstly on the titles and abstracts, followed by examining the methods section of each publication. Here, publications were selected according to pre-defined inclusion and exclusion criteria, and disagreement between reviewers was solved by consensus. Based on the 109 publications included in the original review, a further reduction of titles was undertaken in order to focus on studies addressing walking performance in PwPD, resulting in a total of 34 papers.

### Meta-analysis

The aim of the meta-analysis was two-fold: Firstly, to determine threshold levels of gait variability that discriminates healthy controls (HCs) from PwPD, and secondly to identify whether all variability metrics are indeed elevated in PwPD. In order to achieve this, means and standard deviations (SD) of the various measures of gait variability for both asymptomatic and PwPD cohorts were extracted. In cases where standard error of the mean or 95% confidence intervals (95%CI) were presented, these values were translated into SD as recommended by Cochrane (Higgins, 2011). An effect size (*ES*) for each study was then determined according to Cohen (1988). In addition, each *ES* was corrected for sample size and adjusted to provide Hedges' g (Lipsey and Wilson, 2001). Finally, parameters were grouped to account for different reporting metrics when e.g., coefficient of variation vs. standard deviation of the same parameter was reported. In order to assess the effect of different gait parameter groups in PwPD, a mean *ES* for each gait parameter group was calculated (*ES*′), with studies weighted according to their standard error of measure. Heterogeneity was then assessed using Cochrane's Q and *I*^2^ statistics.

A binary logistic regression (BLR) analysis was then performed on the most commonly reported gait parameter in order to assess how this parameter discriminates the two groups (i.e., HCs and PwPD). The logistic curve-fit was firstly analyzed using the Chi-square goodness-of-fit test, while the quality of the classification was evaluated using a receiver-operating characteristic (ROC) procedure. We then identified the optimal operating point, *y*_*oop*_ with balanced levels of sensitivity as well as specificity. *y*_*oop*_ was then used in an inverse binary logistic regression function in order to assess the optimal threshold value *x*_*oop*_ for the most commonly reported gait measures (Equation 1):

(1) $$\begin{array}{r} \frac{\log_{e}\left(\frac{y_{oop}}{1-y_{oop}}-b_{0}\right)}{b_{1}}=x_{oop} \end{array}$$

## Results

**Figure 1:**
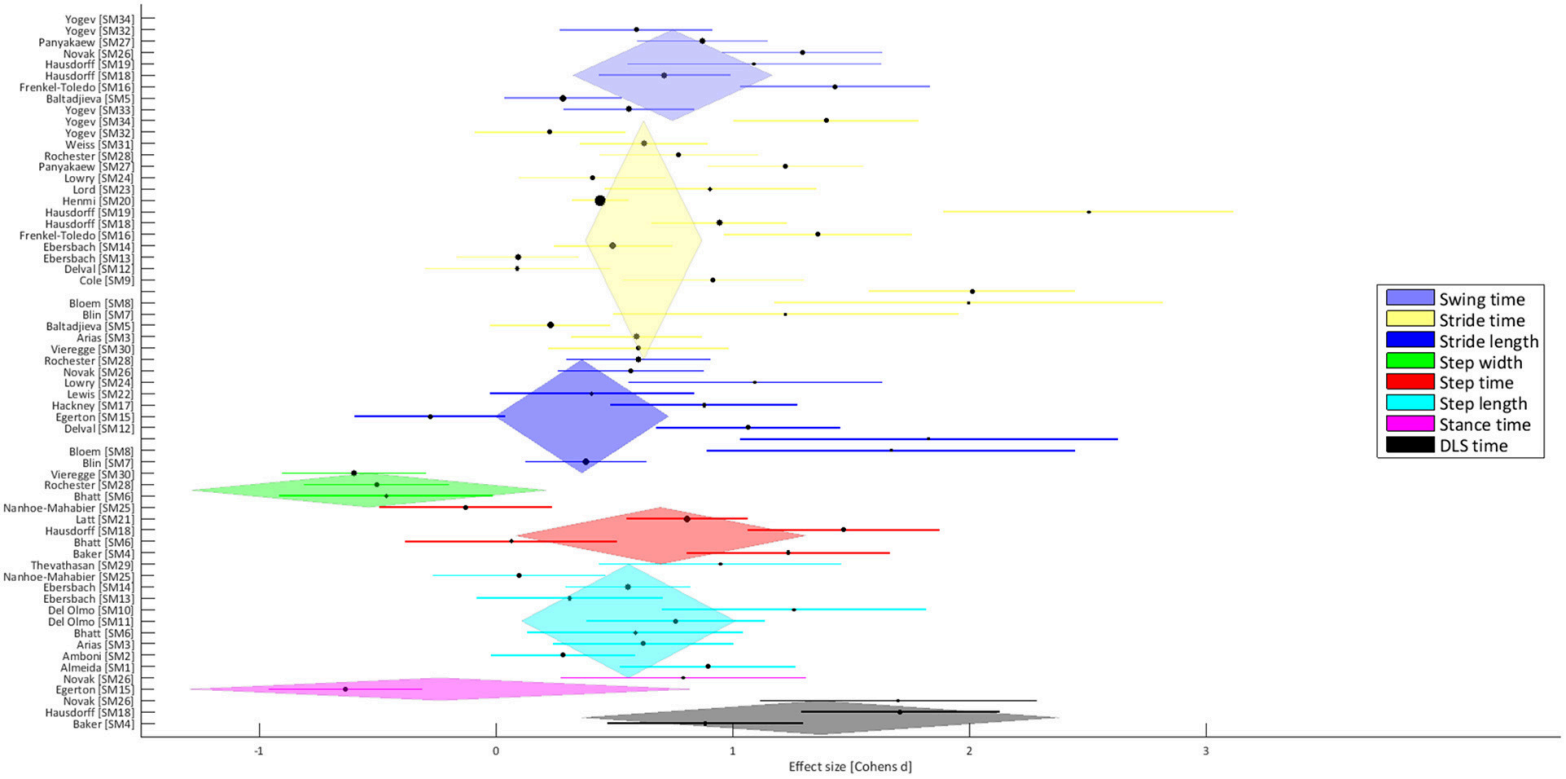
**Forest plot that presents the effect sizes for different gait parameter groups (see Table 1), where a positive ES represents increased variability of PwPD compared to the HCs**.

The 34 publications addressing walking performance in PwPD included a total of 800 PwPD (mean age: 65.6 ± 12.2 years) and 854 HCs (mean age: 65.5 ± 13.2 years). Clinically, disease severity was most commonly evaluated using the Unified Parkinson's Disease Rating Scale (UPDRS part III) (17 studies; range of study means: 6.2 to 50.2), followed by the Hoehn and Yahr scale (9 studies; range of study means: 1.6 to 2.8). Seven studies tested patients in the off-medication condition, one study tested both “on” and “off,” and the remaining studies measured in the on-medication state (electronic Supplementary Table 1). All studies measured subjects during overground walking for the evaluation of gait, most commonly using footswitches (11 studies) followed by pressure sensitive mats (8 studies), but also by using optical motion capture systems (7 studies). Within the 34 publications, some studies reported multiple gait parameters, resulting in a total of 63 reported *ES*-values, with an overall *I*^2^-value of 42.3% and Cochrane's Q of 43.6. There were no significant differences between off-medication and on-medication trials (*ES*′ = 0.71 vs. *ES*′ = 0.75; *p* = 0.94). Eight parameter groups were identified, with variability of stride time (SrT; 21 studies), variability of stride length (SrL; 11 studies) and variability of step length (StL; 10 studies) being the most frequent. The majority of parameter groups showed a positive *ES*′ (ranging from SrL = 0.36 ± 0.19 to variability of double-limb support time = 1.30 ± 0.51), indicating a general increase in gait variability in PwPD compared to HCs (Figure 1). Only the parameter groups of step width variability (StW) (3 studies; *ES* = −0.54 ± 0.38) and stance time variability (SaT) (2 studies; *ES* = −0.24 ± 0.54) revealed a negative *ES*′, indicative of lower levels of variability in PwPD as compared to HCs.

The BLR was conducted on the parameter SrT, including a total of 21 studies with 519 PwPD and 574 HCs, and revealed an area under the ROC curve of 0.74, with a sensitivity of 0.67 and a specificity of 0.78. In the inverse logistic regression, the corresponding *x*_*oop*_ value revealed an optimal coefficient of variation of SrT of 2.4% [95%CI; 1.9 to 3.9] to discriminate Parkinsonian gait from asymptomatic walking.

**Table 1:** **Effect size statistics including the ***z***-test and ***p***-values across all parameter groups**.

	**Swing time**	**Stride time**	**Stride length**	**Step width**	**Step time**	**Step length**	**Stance time**	**Double-limb support time**
Mean effect size	0.75	0.63	0.36	−0.54	0.70	0.56	−0.24	1.30
Mean standard error	0.21	0.13	0.19	0.38	0.31	0.23	0.54	0.51
Comparisons	8	21	11	3	5	10	2	3
*Z*-test	3.48	4.99	1.94	−1.40	2.23	2.43	−0.44	2.66
*p*-value	< 0.1	< 0.1	< 0.1	0.16	< 0.1	< 0.1	0.66	< 0.1

*An alpha level of 10% was used to indicate significant differences in this meta-analysis (Higgins, 2011)*.

## Discussion

Measures of variability have become a popular target for the assessment of gait function in PwPD. However, until now normative values for physiological variability have been missing and it remains unclear how specific gait parameters and their combinations reflect a healthy walking pattern. This systematic review and meta-analysis now provide evidence that 2.4% stride time variability discriminates healthy from pathological walking. More importantly, however, this analysis of the literature has, for the first time, considered how different gait parameters are indicative of pathological disturbances of the HSMS in PwPD. Here, contrary to the common observation of increased levels of variability during gait in these patients, the parameter of step width variability is decreased, hence indicating less flexible motor performance.

The majority of gait parameter groups showed positive ES between PwPD and asymptomatic elderly subjects. This indicates elevated levels of variability during walking in PwPD, which has been associated with reduced walking stability (Dingwell and Kang, 2007; Toebes et al., 2012) as well as a risk factor for falling (Hausdorff et al., 1997; Hamacher et al., 2011; Kobayashi et al., 2014; Konig et al., 2014a), which is a common problem in advanced PwPD cohorts (Wood et al., 2002; Schaafsma et al., 2003; Allen et al., 2011). Here, the parameter of stride time variability in particular has shown to be sensitive in the prediction of falls (Hamacher et al., 2011; Konig et al., 2014a), possibly since variability of temporal gait measures (e.g., stride and step time) depicts the concept of walking rhythmicity, where increased temporal variability is typically associated with observations of unsteady gait (Frenkel-Toledo et al., 2005; Lord et al., 2013). From a neurophysiological perspective, one of the functions of the basal ganglia, in particular the posterior putamen, substantia nigra and globus pallidus, is to maintain rhythmicity during repetitive motor tasks (Plotnik and Hausdorff, 2008; Takakusaki et al., 2008; Lord et al., 2011b; Wu et al., 2015), which is supported by the fact that intervention to these structures in the form of either dopamine replacement therapy or deep brain stimulation has been shown to reverse degenerative changes to temporal variability (Schaafsma et al., 2003; Hausdorff et al., 2009; Bryant et al., 2016). It therefore seems entirely plausible that malfunction or degeneration of specific basal ganglia structures might directly contribute to increased temporal variability during gait in PwPD.

Unexpectedly, two parameter groups showed average negative effect sizes, which is representative of reduced variability during walking in PwPD. While variability of stance time exhibited negative *ES*′, this parameter was only represented by two extremely inconsistent studies and therefore clearly requires further investigation before meaningful conclusions can be drawn. For the parameter of step width variability, however, three studies revealed a highly consistent *ES*′ of −0.54. This indicates that the performance of the patients was less variable, or in other words more rigid, than HCs. Step width variability has been associated with balance performance during walking (Gabell and Nayak, 1984). It is well-established that in PwPD, static as well as dynamic balance is commonly disturbed (Allen et al., 2011; Park et al., 2015; Rinalduzzi et al., 2015) but also that dopamine replacement therapy usually lacks efficacy for reversing the effect (Rocchi et al., 2002; Benatru et al., 2008; Curtze et al., 2015), suggesting that neurophysiological structures other than dopa-sensitive cortico-basal circuits (Takakusaki et al., 2008) (i.e., basal ganglia) are involved in the control of balance during gait (Curtze et al., 2015; Mancini et al., 2015). Here, the pedunculopontine nucleus (PPN) has recently received considerable attention as a major protagonist involved in the control of balance (Hamani et al., 2007; Stefani et al., 2007; Takakusaki et al., 2008; Jahn and Dieterich, 2011; Mancini et al., 2015; Wu et al., 2015). Thus, we hypothesize that the observation of the opposite effect in step width variability in PwPD (i.e., reduced) as compared to parameters of temporal variability, could be explained by the selective effects of pathology on different neurological structures.

From a human movement perspective, however, the observed reduction in step width (spatial) variability, together with an increase in all temporal parameters of variability, might alternatively be explained by a motor compensation mechanism. Here, rather than the direct degeneration of specific neurophysiological structures that exclusively govern particular gait parameters, the loss of control over walking rhythmicity might be actively counter-balanced by tighter regulation of spatial parameters of movement, specifically step width. Here, it should be noted that the greatest effect sizes were observed in the parameter group of double-limb support time (DLS), which is a temporal parameter—therefore affected by disturbances to gait rhythmicity—but one that is also strongly associated with dynamic balance during walking (Lord et al., 2011b). It is therefore conceivable that patients with increased temporal variability compensate by regulating their step width. Such alternative control mechanisms would suggest that balance maintenance is a complex motor function that requires control of both spatial (e.g., step width) and temporal (e.g., DLS and stride time) domains (Todorov and Jordan, 2002), and therefore probably also requires the involvement of different neurophysiological structures. However, further investigation is clearly required to improve our understanding of the relationships between different gait characteristics and their interaction, as well as specific pathophysiology in PwPD.

There are certain limitations to this systematic review of the literature that must be considered when interpreting the presented results. Firstly, the reliability consistency of testing protocols applied in the studies should be considered. Specifically, it has been argued that at least 50 steps are required to ensure reliable measures for walking variability (Konig et al., 2014b), which was only fulfilled in 13 of the 34 (38%) studies. However, testing protocols within a study were similar for both the PwPD and HC groups, and therefore the approaches used possessed equal levels of reliability. Previously, it was shown that low reliability is caused by random error effects and its influence on the derived effect size is therefore negligible (Konig et al., 2014b). However, it is strongly suggested that walking protocols include the assessment of more than 50 steps in future studies. Secondly, a common limitation of reviews is their dependency on publication bias. It seems plausible that results contradicting common expectation on the relationship between pathological and healthy walking performance (i.e., PwPD should exhibit increased variability) are less likely to be published. This results in a relatively higher number of studies presenting positive ESs, which will overestimate the generalized effect of Parkinson's disease on walking performance. For the presented meta-analysis of the literature, such bias could have affected our estimation of the threshold level for stride time variability.

In conclusion, this systematic review and meta-analysis of the literature provides the highest level of evidence for a threshold of 2.4% CV of stride time to discriminate healthy from Parkinsonian gait. Furthermore, it has been shown, that not all gait parameters are equally increased in PwPD. In particular, a decrease in step width variability might be indicative of selective damage to specific neurophysiological structures or alternatively an important compensation mechanism for maintenance of gait stability. Although, the aetiology of disturbed gait characteristics remains to be elucidated, this systematic review is the most comprehensive study to examine the complex interplay between spatial and temporal control in gait in PwPD. However, an accurate analysis of neurophysiological damage in PwPD, together with a reliable and comprehensive assessment walking parameters could lay the foundations for improving our understanding of disturbed walking and control mechanisms during walking in man.

## Author contributions

NK, Data collection and analysis and writing of manuscript. NS, Data collection and analysis and statistical analysis. CB, Interpretation of results and writing of manuscript. WT, Conceptual design of study and writing of manuscript.

### Conflict of interest statement

The authors declare that the research was conducted in the absence of any commercial or financial relationships that could be construed as a potential conflict of interest.
