# Supplementary material for: Can Gait Signatures Provide Quantitative Measures for Aiding Clinical Decision-Making? A Systematic Meta-Analysis of Gait Variability Behavior in Patients with Parkinson's Disease
Source: Front Hum Neurosci. 2016 Jun 30;10:319. doi: 10.3389/fnhum.2016.00319 (PMC4927578; doi:10.3389/fnhum.2016.00319)
Supplement: Supplementary file 1 [file DataSheet1.PDF]

Reference No.	Lead Author	Year	Measurement Technology	Protocol	Estimated number of steps	Cohort	Disease severity		Pathological Cohort				Healthy Cohort			Parameter	Unit	Pathological Cohort		Healthy Cohort	
									Anti-parkinson medication	Sample Size	Gender [m/f]	Age	Sample Size	Gender [m/f]	Age			Mean Variability	SD Variability	Mean Variability	SD Variability
SM1	Almeida	2010	Pressure mat	Overground	10	Basal Ganglia	UPDRS	28.81 ± 6.35	ON	16	10/6	72 ± 6.23	16	6/10	71 ± 5.98	Step Length	CV	0.030	0.020	0.016	0.008
																Gait velocity	m/s	1.073	0.188	1.193	0.128
SM2	Amboni	2012	Optical system	Overground	10	Basal Ganglia	UPDRS III	10.83 ± 5.4	ON	24	20/4	64 ± 6.44	20	10/10	64 ± 3.14	Step Length	CV	3.550	3.500	2.680	2.300
SM3	Arias	2008	Footswitches	Overground	41	Basal Ganglia	H&Y	2.52 ± 0.6	ON	25	15/10	66 ± 7.65	10	na	66 ± 7.65	Stride Time	CV	4.810	4.690	2.330	0.670
																Step Length	CV	6.610	4.880	3.920	1.410
SM4	Baker	2008	Pressure mat	Overground	100	Basal Ganglia	UPDRS	22.9 ± 9.3	ON	14	5/9	70 ± 3.4	12	5/7	72 ± 2.6	Step Time	CV	5.860	1.700	3.970	1.170
																DLS-T	CV	11.460	1.610	9.960	1.680
																Gait velocity	m/s	1.011	0.083	1.273	0.124
SM5	Baltadjieva	2006	Footswitches	Overground	131	Basal Ganglia	H&Y	1.8 ± 0.5	OFF	35	24/11	na ± 13	22	13/9	matched ± 11	Stride Time	CV	2.800	0.900	2.300	0.700
																Swing Time	CV	3.000	1.300	2.400	0.400
																Gait velocity	m/s	1.100	0.210	1.300	0.240
SM6	Bhatt	2013	Optical system	Overground	39	Basal Ganglia	UPDRS III	33.4 ± 10.4	ON	10	na	72 ± 9.8	10	na	70 ± 7.5	Step Length	CV	4.700	2.900	3.300	1.400
																Step Width	CV	12.000	12.800	21.700	25.300
																Step Time	CV	3.400	1.300	3.300	1.600
																Gait velocity	m/s	1.049	0.201	1.443	0.492
SM7	Blin	1990	Wire sensor	Overground	16	Basal Ganglia	H&Y	2.8 ± 1.1	OFF	21	9/12	70 ± 35 *	58	23/35	72 ± 32 *	Stride Length	CV	6.680	4.270	5.170	3.800
																Stride Time	CV	5.510	5.100	4.750	2.300
																Gait velocity	m/s	0.440	0.200	0.830	0.230
SM8	Bloem	1997	Optical system	Overground	25	Basal Ganglia	H&Y	2.3 ± 0.3	OFF	5	1/4	62 ± 4.5	4	3/1	65 ± 5.1	Stride Time	CV	5.090	1.980	2.930	0.710
																Stride Length	CV	14.360	8.290	2.580	0.410
																Gait velocity	m/s	0.820	0.120	1.120	0.060
									ON							Stride Time	CV	4.45	0.65		
																Stride Length	CV	8.8	3.98		
																Gait velocity	m/s	0.98	0.07		
SM9	Cole	2011	Optical system	Overground	39	Basal Ganglia	H&Y	1.6 ± 0.2	ON	17	13/4	66 ± 8.7	15	9/6	68 ± 6.2	Stride Time	ms	28.700	2.560	24.000	1.900
																Gait velocity	m/s	1.150	0.040	1.240	0.050
SM10	del Olmo	2006	Footswitches	Overground	49	Basal Ganglia	UPDRS	29.7 ± 9.8	ON	14	5/4	61 ± 5.2	5	2/3	63 ± 4.3	Gait velocity	m/s	1.180	0.109	1.343	0.083
																Step Length	CV	6.980	3.610	2.760	1.080
SM11	del Olmo	2005	Footswitches	Overground	49	Basal Ganglia	UPDRS	33.8 ± 13.4	ON	15	8/7	62 ± 5.2	15	11/4	63 ± 4.2	Gait velocity	m/s	1.109	0.177	1.285	0.153
																Step Length	CV	7.360	5.230	4.190	2.360
SM12	Delval	2006	Optical system	na	14	Basal Ganglia	UPDRS III	50.2 ± 11.3	OFF	15	na	47 ± 8.7	15	na	50 ± 11.2	Gait velocity	m/s	1.000	0.370	1.360	0.190
																Stride Time	CV	8.660	10.450	1.670	0.640
																Stride Length	CV	8.710	8.680	1.940	1.000
SM13	Ebersbach	1999	Wire sensor	Overground	16	Basal Ganglia	UPDRS III	6.2 ± 4.8	ON	11	3/4	62 ± 10.1	16	7/9	62 ± 8.7	Step Length	CV	2.300	1.300	2.100	1.200
																Stride Time	CV	1.940	0.900	1.800	1.140
SM14	Ebersbach	1999	Wire sensor	na	16	Basal Ganglia	na	na ± na	ON	30	19/11	65 ± 9.3	30	19/11	61 ± 8	Gait velocity	m/s	0.820	0.158	1.032	0.100
																Step Length	CV	3.700	1.900	2.800	1.200
																Stride Time	CV	2.400	0.900	2.300	1.200
SM15	Egerton	2012	Pressure mat	na	20	Basal Ganglia	UPDRS III	14.4 ± 16.7	ON	20	16/4	68 ± 7.9	20	7/13	72 ± 4.1	Stance Time	s	0.018	0.008	0.025	0.013
																Stride Length	cm	2.400	1.200	2.800	1.600
SM16	Frenkel-Toledo	2005	Footswitches	Overground	57	Basal Ganglia	H&Y	2.1 ± 0.2	na	36	na	61 ± 9	30	na	58 ± 7	Gait velocity	m/s	1.120	0.150	1.240	0.180
																Stride Time	CV	2.240	0.740	1.940	0.360
																Swing Time	CV	3.270	1.250	2.800	1.990
SM17	Hackney	2009	Pressure mat	Overground	30	Basal Ganglia	UPDRS motor	27.5 ± 9.2	ON	78	56/22	65 ± 9.5	74	57/17	65 ± 10	Gait velocity	m/s	1.200	0.200	1.200	0.200
																Stride Length	m	0.050	0.006	0.050	0.006
SM18	Hausdorff	1998	Footswitches	Overground	525	Basal Ganglia	na	na ± na	ON	15	10/5	67 ± 36 *	16	2/14	39 ± 54 *	Stride Time	CV	4.400	2.100	2.300	0.500
																Swing Time	CV	7.700	4.100	3.400	0.900
																DLS-T	CV	8.500	1.900	5.800	1.100
																Step Time	CV	5.500	2.400	2.900	0.600
SM19	Hausdorff	2007	Footswitches	Overground	164	Basal Ganglia	H&Y	2.4 ± 0.4	ON	29	15/14	67 ± 9.1	26	12/14	65 ± 6.8	Gait velocity	m/s	1.000	0.210	1.240	0.140
																Stride Time	CV	2.600	1.000	1.800	0.600
																Swing Time	CV	4.700	3.200	2.900	1.300
SM20	Henmi	2009	Accelerometer	Overground	2100	Basal Ganglia	na	na na	ON	9	4/5	66 ± 23 *	10	na	70 ± 16 *	Stride Time	CV	2.380	0.530	1.390	0.140
SM21	Latt	2009	Accelerometer	Overground	33	Basal Ganglia	UPDRS III	12 ± 3	ON	33	5/6	63 ± 11.3	33	5/6	67 ± 11.3	Gait velocity	m/s	1.085	0.135	1.241	0.114
																Step Time	SD	6.147	1.325	4.965	1.561
SM22	Lewis	2000	Optical system	Overground	164	Basal Ganglia	H&Y	2.8 ± 0.8	ON	14	9/5	71 ± 7.6	14	9/5	71 ± 6.5	Stride Length	CV	5.320	2.550	3.420	1.510
SM23	Lord	2013	Pressure mat	Overground	50	Basal Ganglia	UPDRS III	25.4 ± 11.1	ON	122	82/40	67 ± 10.5	184	74/110	70 ± 7.7	Stride Time	SD	0.290	0.130	0.240	0.100
SM24	Lowry	2009	Accelerometer	na	30	Basal Ganglia	MMSE	28.4 ± 1.9	ON	11	8/3	68 ± 7.65	11	8/3	69 ± 8.84	Stride Time	CV	3.900	1.500	2.700	1.000
																Stride Length	CV	3.050	0.880	2.620	1.140
SM25	Nanhoe-Mahabier	2011	Optical system	Overground	79	Basal Ganglia	H&Y	2.1 ± 0.3	OFF	15	11/4	60 ± 35.6	15	9/6	58 ± 28.3	Gait velocity	m/s	1.333	0.231	1.500	0.266
																Step Length	CV	0.033	0.027	0.029	0.049
																Step Time	CV	0.019	0.013	0.023	0.042
SM26	Novak	2006	Footswitches	Overground	630	Basal Ganglia	UPDRS III	18.3 ± 4.7	ON	8	5/3	61 ± 12.4	8	3/5	59 ± 12.3	Gait velocity	m/s	1.020	0.200	1.250	0.200
																Stride Length	CV	5.360	3.100	2.800	0.400
																Stance Time	CV	1.990	1.000	1.290	0.630
																Swing Time	CV	1.860	1.040	0.950	0.400
																DLS-T	CV	2.780	1.600	0.720	0.250
SM27	Panyakaew	2013	Pressure mat	Overground	30	Basal Ganglia	UPDRS III	7.71 ± 3.9	OFF	21	na	64 ± 9.41	21	na	62 ± 8.04	Gait velocity	m/s	1.065	0.140	1.060	0.181
																Swing Time	CV	4.440	1.180	3.200	0.610
																Stride Time	CV	2.550	0.780	2.230	0.760
SM28	Rochester	2012	Pressure mat	Overground	50	Basal Ganglia	UPDRS III	29.14 ± 9.54	ON	22	16/6	70 ± 9.67	22	9/13	67 ± 8.43	Gait velocity	m/s	1.120	0.240	1.310	0.150
																Stride Length	m	0.037	0.013	0.030	0.011
																Stride Time	s	0.032	0.013	0.019	0.007
																Step Width	cm	1.930	0.620	2.230	0.550
SM29	Thevathasan	2012	Pressure mat	Overground	40	Basal Ganglia	UPDRS III (1-26)	29.4 ± 9.5	OFF	8	5/3	64 ± 6.1	9	7/2	67 ± 8.3	Step Length	CV	0.030	0.010	0.020	0.010
SM30	Vieregge	1997	Paper walkway	Overground	40	Basal Ganglia	UPDRS III	37.5 ± 16.8	ON	17	11/6	69 ± 7.4	33	17/16	70 ± 7	Gait velocity	m/s	0.980	0.190	1.270	0.180
																Stride Length	CV	5.200	6.600	2.800	1.100
																Step Width	CV	22.700	11.200	26.900	2.900
SM31	Weiss	2011	Accelerometer	Overground	105	Basal Ganglia	UPDRS Motor	23.6 ± 9.4	OFF	22	na	66 ± 5.9	17	na	70 ± na	Stride Time	CV	1.980	0.710	1.490	0.480
SM32	Yogev	2005	Footswitches	Overground	210	Basal Ganglia	UPDRS III	17.5 ± 8.3	ON	30	na	71 ± 7.9	28	na	70 ± 6.3	Gait velocity	m/s	1.050	0.230	1.310	0.190
																Stride Time	CV	2.110	0.730	1.720	0.460
																Swing Time	CV	2.760	1.360	1.860	0.400
SM33	Yogev	2013	Footswitches	Overground	105	Basal Ganglia	UPDRS III	23.3 ± 9.1	ON	18	na	69 ± 4.7	15	/	75 ± 5.4	Gait velocity	m/s	1.180	0.160	1.320	0.160
																Stride Time	CV	1.450	0.330	1.060	0.180
SM34	Yogev	2012	Footswitches	Overground	105	Basal Ganglia	UPDRS III	20.7 ± 8.9	ON	20	12/8	70 ± 6.6	20	10/10	71 ± 7	Gait velocity	m/s	1.190	0.150	1.390	0.220
																Stride Time	CV	1.930	0.570	1.800	0.540
																Swing Time	CV	2.970	0.800	2.520	0.680

*	Deviation measure is range
@	Deviation measure is interquartile range
§	Median & Range
#	Median & Interquartilerange
